# STRATEGIES TO ENSURE AUTHENTIC PARTICIPANTS AND VALID DATA WITH ONLINE RECRUITMENT OF FAMILY CAREGIVERS

**DOI:** 10.1093/geroni/igac059.2411

**Published:** 2022-12-20

**Authors:** Henrietta Armah, Jasmine Vickers, Danny Wang, Carolyn Pickering

**Affiliations:** University of Alabama at Birmingham, Birmingham, Alabama, United States; University of Alabama at Birmingham, Birmingham, Alabama, United States; The University of Alabama at Birmingham, Birmingham, Alabama, United States; The University of Alabama at Birmingham, Birmingham, Alabama, United States

## Abstract

Over the past years, recruitment of participants for behavioral and biomedical research through the internet has become more popular. Online has become an advantageous approach to recruitment, especially since the covid pandemic has posed a great challenge to most in-person research activities. Typically, internet-based recruitment strategies include website posting, emailing list of potential participants as well as using social media such as Facebook, Twitter, and Instagram to deliver recruitment information to groups who are often unrepresented in research. Although this mechanism has reduced barriers to participation, it has posed serious threats to data quality and validity. For example, some studies on data validity estimate that up to 90% of online survey responses are fraudulent when they rely on screening questions and CAPTCHA alone. Others have shown that vetted panel data such as Mechanical Turk (mTurk), has high rates of participant misrepresentation. Therefore, the aim of this paper is to highlight the challenges associated with internet-based recruitment of family caregivers and describe strategies for researchers to ensure data integrity. We discuss multi-faceted approaches to detect and prevent fraudulent and suspicious activities such as duplicate and automated enrollment by software applications known as bots as well by fraudulent human participants. We discuss data on several strategies that have proven effective in our previous and ongoing trails. We will also demonstrate the need to implement several strategies and a “fail-safe” to detect fraud after enrollment. It is imperative that researchers understand the need to address these challenges to preserve data integrity and replicability.

